# Is Lethality Different between Males and Females? Clinical and Gender Differences in Inpatient Suicide Attempters

**DOI:** 10.3390/ijerph192013309

**Published:** 2022-10-15

**Authors:** Isabella Berardelli, Elena Rogante, Salvatore Sarubbi, Denise Erbuto, Mariarosaria Cifrodelli, Cristina Concolato, Massimo Pasquini, David Lester, Marco Innamorati, Maurizio Pompili

**Affiliations:** 1 Department of Neurosciences, Mental Health and Sensory Organs, Faculty of Medicine and Psychology, Suicide Prevention Centre, Sant’Andrea Hospital, Sapienza University of Rome, Via di Grottarossa 1035, 00189 Rome, Italy; 2 Department of Human Neurosciences, Sapienza University of Rome, Viale dell’Università 30, 00185 Rome, Italy; 3 Psychiatry Residency Training Program, Psychiatry Unit, Faculty of Medicine and Psychology, Sant’Andrea Hospital, Sapienza University of Rome, Via di Grottarossa 1035, 00189 Rome, Italy; 4 Psychology Program, Stockton University, Galloway, NJ 08205, USA; 5 Department of Human Sciences, European University of Rome, Via degli Aldobrandeschi 190, 00163 Rome, Italy

**Keywords:** suicide, gender, prevention, males, females

## Abstract

According to the gender paradox in suicidology, an important sex difference has been reported with a preponderance of females in nonfatal suicidal behavior and a preponderance of males in completed suicide. Furthermore, females and males present different risk factors for suicide. The present study explored possible clinical differences between male and female psychiatric inpatients who had recently attempted suicide. The study included 177 adult inpatients hospitalized following a suicide attempt at the University Psychiatric Clinic, Sant’Andrea Hospital, Sapienza University of Rome. Clinical features assessed included psychiatric diagnosis, method and lethality of suicide attempts using the Risk/Rescue Rating Scale, the history of suicide attempts, age at onset of psychiatric illness, the presence of substance or alcohol use, and the length of stay. The results found that males and females differed in the method used for the suicide attempt, the scores for risk and rescue, and the length of hospitalization post-suicide attempt. In conclusion, identifying gender characteristics of patients at higher risk of suicide is important for implementing specific suicide prevention strategies and reducing the risk of future suicidal behavior in psychiatric inpatients.

## 1. Introduction

The need to identify patients at a higher risk for attempting suicide is a clinical challenge and has stimulated researchers to better investigate the clinical and demographical features of patients who attempt suicide [1,2], because a suicide attempt strongly predicts future suicide attempts [3,4,5]. Suicide attempts, however, occur 15–20 times more frequently than completed suicides, causing important physical and psychological damage for the patient [6].

Gender differences in mental health are among the most interesting findings in psychiatry including differences regarding prevalence, symptomatology, risk factors and the course of the psychiatric disorder [7]. According to the gender paradox in suicidology, an important sex difference has been reported for suicidal behavior. Females have higher rates of suicide ideation and suicide attempts than males, while fatal suicide acts are typically higher for males than for females [8,9,10,11,12]. The lethality of suicidal behavior in females is lower, most likely because males choose more violent suicide methods [8,13,14]. However, the reason why males and females choose different suicide methods is still controversial. One hypothesis is that men tend to gravitate toward more lethal methods than females because they have higher suicide intent [15,16,17,18]. Canetto et al. [8] proposed that women and men use different suicide methods as a result of the sex roles in their culture.

From an epidemiology point of view, suicide rates in women and men often depend on other features including age and ethnicity [19,20]. Furthermore, suicide rates vary significantly between countries and over time, and studies on gender differences across countries are still controversial [21,22,23,24,25]. Interestingly, women more frequently present traditional risk factors for suicide than men, including depression [26], childhood sexual abuse [27], and prior suicidal ideation and attempts [28].

Hitherto, only a few studies have investigated the clinical profiles of men and women who attempt suicide [29,30,31,32]. Monnin et al. [29] in a study on 273 participants selected in psychiatric emergency units after a suicide attempt, observed that women presented a higher rate of anxiety disorders and men presented more alcohol dependence and drug dependence and abuse. Furthermore, the authors described gender differences in the suicidal act; women presented more impulsivity and men had a higher suicidal intent [29]. However, a comprehensive review on the role of sex in self-injurious thoughts and behaviors showed that studies on the differences in suicide intent across suicide attempts in males and females is mixed, with some studies showing that males report higher suicide intent, and others not showing a significant difference [31]. Sex differences and a change in the lethality of suicidal behaviors were reported by Isometza and Lonnqvist [33]. Death by suicide is influenced by the chances of rescue during the suicide attempt [34,35,36] and depend on factors such as the availability of the method preferred and chosen for suicide, knowledge of lethal effects of the method (e.g., how many pills to take), familiarity with using a particular method (e.g., familiarity with firearms), and the concomitant use of alcohol, drugs and medications [37,38]. In their study, Berardelli et al. [39] assessed suicide method lethality in 107 psychiatric inpatients who attempted suicide and found that patients who used a highly lethal method presented specific demographical and clinical characteristics, but they found no differences by gender [39]. Other studies showed that men are more likely to use relatively more lethal methods than women [8,10]. Particularly, firearms and jumping in front of moving objects were observed frequently in males, and self-poisoning in females [29,40].

Based on the hypothesis that females and males present different risk factors for suicide [28], the purpose of the present study was to explore possible clinical differences between male and female psychiatric inpatients who had recently attempted suicide. We studied patients hospitalized in a psychiatric unit soon after a suicide attempt. Clinical features assessed included psychiatric diagnosis, the methods and lethality of the suicide attempt, the history of suicide attempts, age at onset of psychiatric illness, the presence of substance or alcohol use, and the length of stay. We hypothesized that these clinical characteristics would differ between male and female suicide attempters. In particular, we expected that females used less lethal methods for their suicide attempt and obtain lower scores for risk and rescue. We also expected differences in the length of stay, age, diagnosis, history of suicide attempt, age at onset and substance and alcohol use.

## 2. Materials and Methods

### 2.1. Participants

The present study included 177 adult inpatients consecutively enrolled at the University Psychiatric Clinic, Sant’Andrea Hospital, Sapienza University of Rome hospitalized following a suicide attempt between January 2018 and May 2022. The sample consisted of 74 males and 103 females. The mean age of the participants was 40.46 years (standard deviation = 16.13; age-range = 18–81 years). The sociodemographic and clinical characteristics of the sample are summarized in Table 1. Inclusion criteria were a current suicide attempt requiring hospitalization, adult inpatient aged ≥ 18 years and informed consent for participation in the study. Exclusion criteria was the presence of severe neurological disorders (epilepsy, cognitive impairment, or genetic syndromes) or the presence of cognitive deficits causing linguistic and comprehension problems.

The assessment of psychiatric patients, with particular attention to suicide risk, is part of several investigations approved by the local ethics review board of Sant’Andrea Hospital—Sapienza University of Rome. In addition, the study analyzed the demographical and clinical characteristics of patients as part of a broader investigation approved by the local Institutional Review Board of Sant’Andrea Hospital—Sapienza University of Rome. The study was conducted according to the guidelines of the Declaration of Helsinki.

### 2.2. Measures

Three independent psychiatrists at the University Psychiatric Clinic, Sant’Andrea Hospital, Sapienza University of Rome, analyzed the clinical record of each patient. Data were collected using a structured checklist created specifically for this study. In the checklist, researchers included information about psychiatric diagnosis, the method used for attempting suicide, the history of suicide attempts, age at onset of psychiatric illness, the presence of substance or alcohol use, and the length of stay.

Psychiatric diagnosis was performed during the first days of hospitalization, within 48 hours of the admission, based on the Diagnostic and Statistical Manual of Mental Disorders, fifth edition [41] and supported by the Structured Clinical Interview for DSM-5 Disorders [42].

Trained psychiatrists assessed the suicide attempt when the patient arrived at the emergency department. Suicide attempt was assessed referring to the definition provided by Silverman et al. [43,44] in which type II suicide attempts are described as self-destructive acts with some degree of intent to end one’s life and some identifiable injuries. For this study, suicide attempt was considered as present if it was the reason for the hospitalization.

The Risk/Rescue Rating Scale [35] was used to assess the lethality of the current suicide attempt. The scale consists of 10 items: 5 risk factors (i.e., method used, toxicity, reversibility, treatment required, impaired consciousness), and 5 rescue factors (i.e., delay until discovery, the person initiating rescue, probability of discovery, location, accessibility to rescue). Each item is scored on a scale ranging from 1 to 3, and the total risk and rescue subscale scores are classified into five categories for both risk and rescue factors (from 1, low risk, to 5, higher risk, and from 1, least rescuable, to 5 most rescuable, respectively.) Thus, the risk and the rescue scores range from 1 to 5. A higher risk score indicates a more lethal suicide attempt, while a higher rescue score indicates a more rescuable suicide attempt.

### 2.3. Statistical Analysis

All statistical analyses were performed with the Statistical Package for Social Sciences (SPSS 27.0, SPSS Inc., IBM, Chicago, IL, USA). A series of *t* tests, Fisher exact tests, and chi-squared (χ²) tests were used for bivariate analyses. In cases of non-normal distribution, the Mann–Whitney test was used. Variables significant at the bivariate analyses were then included as independent variables in a binary logistic regression model, with sex as dependent variable. For the regression model, multicollinearity was checked through the variance inflation factor (VIF) The Hosmer–Lemeshow goodness-of-fit test (HL) was used to examine whether the model fit the data adequately; when the *p*-value is > 0.05, it usually means a good fit. All tests were considered statistically significant for *p*-values < 0.05.

## 3. Results

### 3.1. Sample Characteristics

There were 52 patients (29.4%) with a personality disorder, 38 (21.5%) with depressive disorder, 31 (17.5%) with bipolar disorder, and 28 (15.8%) with schizophrenia or other psychotic disorders. The mean age at onset was 28.61 (SD = 15.93). Twenty-nine patients (16.4%) reported substance use, while twenty-five patients (14.1%) reported alcohol use. Seventy-seven patients (44.5%) had a history of suicide attempt. The mean length of stay was 9.26 days (SD = 7.27). The commonest method used for current suicide attempt was drug/poison ingestion (63.8%), followed by jumping (13.6%), hanging (12.4%), and cutting (10.2%). According to the Risk/Rescue Rating Scale, the mean score for the risk scale was 3.60 (SD = 0.71) while the mean score for the rescue scale was 2.64 (SD = 1.15) (Table 1).

### 3.2. Differences between Groups

Males and females differed according to the method used for suicide attempt (χ²_3_ = 10.96, *p* < 0.05), the scores for risk and rescue (t_175_ = 2.55, *p* < 0.05; t_146.6_ = −1.99, *p* < 0.05, respectively), and the length of stay (U = 3084.5, *p* < 0.05). The groups did not differ in terms of age, diagnosis, history of suicide attempt, age at onset, and substance and alcohol use (Table 1).

Females were more likely to use drug/poisoning ingestion as method for suicide attempt than males (72.8% vs. 51.4%), whereas males were more likely to use hanging than females (20.3% vs. 6.8%). The risk score was higher for males (3.76 ± 0.68) than for females (3.49 ± 0.72), and the rescue score was higher for females than for males (2.79 ± 1.09 vs. 2.43 ± 1.22). Finally, the length of stay was longer for males than for females (10.66 ± 8.09 vs. 8.25 ± 6.48).

In this study, VIF values were around 1, which indicated that multicollinearity was not an issue. A binary logistic regression model (not reported in the tables) with sex as the criterion and significant variables at the bivariate analysis inserted as independent variables explained about 14% of the variance in the data (Nagelkerke R^2^ = 0.144; −2LL = 219.603; χ^2^_6_ = 19.91, *p* < 0.01). Since the HL test was significant the following results should be read with caution. Compared to males, females were more likely to use drug/poisoning ingestion as method for suicide attempt (χ^2^ = 8.33, *p* < 0.01; OR = 5.05, 95% C.I. = 1.52/16.68), whereas males had a higher level of risk score (χ^2^ = 6.08, *p* < 0.05; OR = 0.57, 95% C.I. = 0.33/0.99), and a longer length of stay (χ^2^ = 4.76, *p* < 0.05; OR = 0.95, 95% C.I. = 0.91/0.99) than females.

## 4. Discussion

The purpose of the present study was to identify possible clinical factors that may predict future suicide attempts in a sample of male and female psychiatric inpatients. who had recently attempted suicide.

Regarding the method used for suicide attempt, our results showed that females were more likely to use drug/poisoning ingestion as method for suicide attempt than males (72.8% vs. 51.4%), whereas males were more likely to use hanging than females (20.3% vs. 6.8%). These results confirm other studies on the role of difference in suicide methods used by males and females for explaining and supporting the “gender paradox” hypothesis. Previous research has shown that men are more likely to use relatively more lethal methods than women [8,10,11,45]. In contrast, women are more likely to attempt suicide using poisoning [13], a method which allows more time for intervention by others. These differences in the choice of suicide method could also influence official statistics on suicide. Several studies have suggested that female fatal suicides are under-reported as compared with those of men [8,10,46,47], because not all the drug deaths are identified as suicides. Conversely, the violent methods often used by men can rarely be classified as accidental deaths.

The lethality of a suicide attempt can be expressed as a ratio of factors influencing risk and rescue. Risk/Rescue Rating Scale scores [35] showed that the risk score was higher in males (3.76 ± 0.68) than in females (3.49 ± 0.72), and the rescue score was higher in females than in males (2.79 ± 1.09 vs. 2.43 ± 1.2). These results can be explained by the fact that, since women are more likely to attempt suicide by poisoning which can take a long time before death occurs (and for which it is also difficult to calculate a lethal dose), they have a greater chance of being rescued than do men who usually choose more lethal methods. Why women choose less lethal means than men have been attributed to several factors, including their lesser intent to die, gender socialization, and the easy availability of methods to women and to men. As reported in a previous paper on this topic [39], factors which influence lethality of suicidal acts not only depend on the choice of the methods but also other factors such as the social and communicative context of the suicidal act, the social acceptability of behaviors, learning from models (cognitive availability), and ease of access to or technical ability regarding the chosen suicide method [48]. Knowledge of and access to firearms is probably easier for males than for females, and females may be more concerned with preserving their appearance, trying to ensure that their body and face are not severely injured [13,49]. The Interpersonal Theory of suicide may also contribute to explaining sex differences in suicidal behavior [50,51]. The capability for suicide may be more relevant for men than for women since men are more likely to have had experiences which habituate them to pain (such as experience in the military and violent sports). In fact, several studies reported that men do experience greater self-reported suicide capability [52,53,54,55] and pain tolerance [56,57].

The two groups did not differ in terms of age, diagnosis, history of suicide attempt, age at onset, or substance and alcohol use. However, the length of stay of the hospitalization was longer for the male patients than for the female patients, suggesting a greater clinical complexity for the male patients who had attempted suicide and a need for a longer hospitalization for the stabilization of clinical conditions, the setting of their pharmacotherapy and the prevention of future suicide attempts. In fact, suicide attempts that lead to hospital admission are associated with a higher risk for future suicide attempt, with the greatest risk in the initial months after discharge [58]. Several factors are associated with a more severe clinical complexity of male psychiatric patients including less prior use of health care services for routine and yearly visits and less use of prevention services [59,60,61]. Male patients are also less likely to comply with medical recommendations [59]. 

The present study has several limitations. First, our patient sample was relatively small, and the patients had different psychiatric disorders. Hospitalized patients may have had several admissions before the index evaluation performed in this study, and they may have had a complicated clinical history of pharmacological treatment. These factors may limit the study’s generalizability. The cross-sectional and retrospective nature of the study design should be considered an additional drawback. In this study, the main aim was to investigate clinical differences in suicide attempts in male and female inpatients, and we did not investigate suicidal intent. Furthermore, we did not administer tools assessing toxicity in the patients with drug overdoses, and we did not assess the number of, and methods used in previous suicide attempts. Finally, we did not use psychometric tools evaluating the severity of psychiatric symptoms or the presence of other psychopathological features involved in the patients.

## 5. Conclusions

In conclusion, the results of this study have important implications for possible suicide prevention strategies in patients with psychiatric disorders. The present study illustrates the usefulness of the Risk/Rescue Rating Scale [35], which is a descriptive and quantitative method of assessing the lethality of suicide attempts. More lethal attempts at suicide are most likely stronger predictors of future death by suicide. Identifying sex-related characteristics of suicide risk in patients is important for implementing specific suicide prevention strategies to reduce suicidal intent, psychological pain, and rehospitalization in patients with psychiatric disorders. Men and women may need different strategies for the prevention of future suicidal behavior. The complexity of this phenomenon lies in the unmet needs of suicidal individuals who may not ft into diagnostic categories and may lack a full clinical picture. Therefore, it is important that modern psychiatry tries to explain suicide risk: not reducing it to a symptom, but considering the unfortunate combination of factors that threaten the stability of an individual [62].

## Figures and Tables

**Table 1:** Differences between groups.

Variable	Whole Sample (*N* = 177)	Males (*N* = 74; 41.8%)	Females (*N* = 103; 58.2%)	Test	Significance
Age (M ± SD)	40.46 ± 16.1	41.73 ± 14.6	39.55 ± 17.1	t_169.9_ = 0.91	0.365
Diagnosis				χ²_4_ = 3.25	0.518
Bipolar disorder	31 (17.5)	10 (13.5)	21 (20.4)		
Depressive disorder	38 (21.5)	16 (21.6)	22 (21.4)		
Schizophrenia spectrum or other psychotic disorders	28 (15.8)	13 (17.6)	15 (14.6)		
Personality disorder	52 (29.4)	20 (27.0)	32 (31.1)		
Other	28 (15.8)	15 (20.3)	13 (12.6)		
Age at onset (M ± SD)	28.61 ± 15.9	31.00 ± 15.1	26.89 ± 16.4	t_168_ = 1.67	0.097
History of suicide attempt	77 (44.5)	31 (42.5)	46 (46.0)		0.757 ^a^
Substance use	29 (16.4)	16 (21.6)	13 (12.6)		0.149 ^a^
Alcohol use	25 (14.1)	14 (18.9)	11 (10.7)		0.131 ^a^
Length of stay (days; M ± SD)	9.26 ± 7.3	10.66 ± 8.1	8.25 ± 6.5	U = 3084.5	0.038 *
Method used for suicide attempt				χ²_3_ = 10.96	0.012 *
Cut/pierce	18 (10.2)	8 (10.8)	10 (9.7)		
Drugs/poison ingestion	113 (63.8)	38 (51.3)	75 (72.8)		
Jumping	24 (13.6)	13 (17.6)	11 (10.7)		
Hanging	22 (12.4)	15 (20.3)	7 (6.8)		
RRS—Risk score	3.60 ± 0.71	3.76 ± 0.68	3.49 ± 0.72	t_175_ = 2.55	0.012 *
RRS—Rescue score	2.64 ± 1.15	2.43 ± 1.2	2.79 ± 1.09	t_146.6_ = −1.99	0.048 *

^a^ = Fisher’s exact test. * = *p* < 0.05.

## Data Availability

The data presented in this study are available on request from the corresponding author.
